# Highly effective isolation of *Stenotrophomonas* from pharyngeal swabs of infected inpatients and issues in species identification

**DOI:** 10.1128/spectrum.03798-25

**Published:** 2026-02-25

**Authors:** Boqing Xu, Yawei Zeng, Zelin Yu, Yi Jiang, Yanyan Zhou, Chao Wang, Junyi Liu, Ruibai Wang, Wenjie Qi

**Affiliations:** 1 National Key Laboratory of Intelligent Tracking and Forecasting for Infectious Disease, Chinese Centre for Disease Control and Prevention, National Institute for Communicable Disease Control and Prevention 96698, Beijing, People's Republic of China; 2 Beijing Friendship Hospital, Capital Medical University 12517, Beijing, People's Republic of China; Huashan Hospital of Fudan University, Shanghai, China

**Keywords:** *Stenotrophomonas*, inpatient, species, average nucleotide identity, MALDI-TOF, identification

## Abstract

*Stenotrophomonas* is a significant opportunistic pathogen associated with high attributable mortality. Notably high isolation rates and species diversity have been observed among tuberculosis patients in Beijing, China. To determine its infection/colonization rate and characteristics in non-TB patients, we conducted a study utilizing a sensitive pre-enrichment PCR assay on pharyngeal swabs from inpatients in the Department of Infectious Diseases. Three *Stenotrophomonas* strains were isolated from 143 samples (2.10%), and positive cases shared common characteristics—advanced age, invasive procedures, repeated hospitalizations, and broad-spectrum antimicrobial exposure—which are established risk factors for *Stenotrophomonas maltophilia* infection. Both the isolation rate and common characteristics differed from those of TB patients. Notably, three isolates were identified via average nucleotide identity (ANI) as *S. geniculate* and *S. muris*, but both species were misidentified as *S. maltophilia* by matrix-assisted laser desorption/ionization time-of-flight (MALDI-TOF) mass spectrometry, despite achieving reliable identification scores (≥2.0). This misidentification likely explains the exclusive focus on *S. maltophilia* and the underestimation of other species absent from MALDI-TOF databases. Antibiotic susceptibility testing showed general susceptibility to quinolones, tigecycline, chloramphenicol, and trimethoprim-sulfamethoxazole and partial susceptibility to ceftazidime and ceftazidime/avibactam. Notably, a strain susceptible to ceftazidime and moxifloxacin was isolated from a patient treated with these agents. In conclusion, this study underscores the necessity of developing alternative rapid molecular methods with ANI-equivalent resolution for *Stenotrophomonas*, alongside expanding mass spectral databases and reevaluating the clinical significance and resistance profiles of the *Stenotrophomonas* species.

**IMPORTANCE:**

Compounding its inherent pathogenicity, *Stenotrophomonas* adversely impacts co-infecting pathogens and is associated with high attributable mortality and multidrug resistance. This study demonstrates that through enrichment and PCR of throat swab samples, this elusive pathogen—frequently undetected in routine clinical testing—can be identified with high sensitivity, providing crucial diagnostic alerts for clinicians, particularly in critically ill and etiology-unknown patients. Our analysis reveals that the risk factors for *Stenotrophomonas* positivity differ between general infectious disease inpatients and tuberculosis patients. Furthermore, our findings highlight a critical issue of species misidentification by routine microbiological methods, which has obscured the true clinical significance and resistance profiles of *Stenotrophomonas* species.

## INTRODUCTION

*Stenotrophomonas* is a gram-negative bacterium genus of the family *Lysobacteraceae* that is widely present in natural environments. In the systematic classification based on the average nucleotide identity (ANI) of whole genomic sequences, the *Stenotrophomonas* genus comprises 116 ANI genotypic species, among which 32 species have been validly named (1). Besides their applications in industry and agriculture as plant growth-promoting rhizobacteria, emerging biodegradation agents, and substitutes for synthetic fungicides, *Stenotrophomonas* strains represented by *Stenotrophomonas maltophilia* are important clinical opportunistic pathogens and can cause infections in various tissues and organs (2–4). The three key characteristics of *Stenotrophomonas* are multidrug resistance, high mortality rate, and a strong ability to form biofilms (5, 6). The attributable mortality rate of *S. maltophilia* infection ranges from 24% to 58%, with the mortality rate of bacteremia as high as 65%, far exceeding that of carbapenem-resistant *Acinetobacter baumannii* (42%) (7). *S. maltophilia* infection has been recognized as a life-threatening disease in ICU patients, cancer patients, and immunocompromised individuals, as well as a marker of pulmonary function deterioration. The World Health Organization has classified it among the globally most concerning emerging multidrug-resistant (MDR) bacteria (8). The Infectious Diseases Society of America has included *S. maltophilia* in its guidelines for the treatment of drug-resistant infections, alongside extended-spectrum β-lactamase-producing *Enterobacterales*, carbapenem-resistant *Enterobacterales*, *Pseudomonas aeruginosa*, ampC β-lactamase-producing *Enterobacterales*, and carbapenem-resistant *A. baumannii*. This highlights the global risks of these severe infections with high morbidity and mortality, as well as the challenges they pose to clinical management (9).

The 2024 data from the China Antimicrobial Surveillance Network reveal that *S. maltophilia* ranks ninth among clinically isolated bacterial species across all sample types, with an isolation rate of 2.8%. When focusing specifically on respiratory tract samples, this bacterium places eighth, and its isolation rate rises to 5.2%. However, our previous study showed that the crude isolation rate of *Stenotrophomonas*, based solely on culture method in sputum samples from tuberculosis (TB) patients in a TB-specific hospital in Beijing, reaches 10.11%. We also identified two known *Stenotrophomonas* species (*S. maltophilia* and *S. rhizophila*) as well as two novel species, namely *S. pigmentata* and *S. tuberculopleuritidis* (10). To determine whether TB patients have a higher infection/colonization rate of *Stenotrophomonas* than other patients and different characteristics, in this study, we chose a large-scale comprehensive hospital in Beijing as a comparison and collected the pharyngeal swab samples from the inpatients in its infectious diseases department, which generally have more severe clinical conditions and receive more antibiotic treatments, to detect and isolate *Stenotrophomonas* strains. Moreover, our previous study revealed that a considerable proportion (45.34%) of *S. maltophilia* strains were misidentified; they actually belong to other species within the *S. maltophilia* complex (Smc) or even more distantly related species (1). This issue has also been explored in this study.

## MATERIALS AND METHODS

### Sample collection

Pharyngeal swab samples were collected from inpatients in the Department of Infectious Diseases of Beijing Friendship Hospital, Capital Medical University, from January to April 2025.

### Preparation of simulated samples and detection efficiency evaluation

Fresh culture of the established Chinese *S. maltophilia* reference strain 11066 (CHPC 1.13916) (11) was used to prepare 1 McFarland bacterial suspension, which was then subjected to 10× serial dilution using phosphate-buffered saline (PBS). For each dilution, 1 mL of the bacterial solution was aliquoted into two 1.5 mL Eppendorf centrifuge tubes and centrifuged at 10,000 × *g* for 5 min. The pellets from one series of dilutions were resuspended in 50 μL of PBS. The pellets from the other series were resuspended in 1 mL of Luria-Bertani (LB) liquid medium and incubated at 37°C for 1 day, then centrifuged again at 10,000 × *g* for 5 min; the resulting pellet was resuspended in 50 μL of PBS. Meanwhile, colony counting was performed on the prepared 1 McFarland turbidity bacterial suspension to obtain the actual bacterial concentration.

### Enrichment culture and species identification

After sampling, the swabs were thoroughly immersed in 3 mL of PBS. Two milliliters of the swab liquid was centrifuged at 10,000 × *g* for 5 min. The pellet was resuspended and enriched in 1 mL of LB liquid medium. Alternatively, 2 mL of the swab liquid was mixed with 2 mL 2× LB liquid, then incubated at 37°C for 1 day. Cultures with positive PCR results were streaked onto LB agar plates using the three-section method. After 2 days of incubation at 37°C, single colonies were picked for another round of PCR verification. Positive colonies were cultured with LB broth and stored.

### PCR amplification

Boiled DNA templates from the fresh cultures of the tested strains were used in PCR. The 20 μL PCR mixture was formulated as follows: 1 μL DNA template, 10 μL 2× EasyTaq PCR SuperMix (TransGen Biotech, Beijing, China), 1 μL of 10 μM of each primer, and 8 μL purified water. PCR amplification condition was 5 min at 95°C followed by 30 cycles of 95°C for 30 s, 58°C for 30 s, and 72°C for 1 min, with a final extension step at 72°C for 5 min. The primers used include *Stenotrophomonas*-specific primers: guaA-forw: AAC GAA GAA AAG CGC TGG TA and guaA-rev: ACG GAT GGC GGT AGA CCA T; and 16S rRNA universal primers: 16S-U: AGA GTT TGA TCM TGG CTC AG and 16S-L: CCG TCA ATT CMT TTR AGT TT.

### Next-generation sequencing and ANI calculation

Genomic DNA of the strains was extracted using the QIAamp DNA Mini kit (51304; QIAGEN GmbH, Germany). Next-generation whole-genome sequencing was conducted by Shanghai Majorbio Biomedical Technology Co., Ltd. The strain’s species was identified by calculating its ANI with all of the 116 ANI species reference genomes of the genus *Stenotrophomonas* using skani (https://github.com/bluenote-1577/skani) (1). Phylogenetic trees were constructed using IQtree (v2.3.6) (https://github.com/iqtree/iqtree2) (12) with automatic model selection and 1,000 bootstrap replicates, and visualized using Tree Visualization By One Table (13). *Xanthomonas campestris* genome (MAFF106181, GCF_013388375.1) was used as an outgroup.

### Genomic pairwise difference analysis

Whole-genome alignment of the assembled sequences from strains within the same species was performed using MUMmer (v3.07). The nucmer program was executed to produce a delta file, which was subsequently processed with delta-filter to remove redundant and low-identity short alignments. Single-nucleotide polymorphisms (SNPs) and insertions/deletions (InDels) were then identified from the filtered delta file using the show-SNPs utility.

### Antibiotic susceptibility testing

The customized antibiotic susceptibility testing (AST) plate for Chinese Pathogen Identification Net (CHNENF; Trek Diagnostic Systems Ltd, West Sussex, United Kingdom) was used to determine the strain’s sensitivity to 17 drugs commonly used for gram-negative strains. Judgment was made according to the breakpoints for *Stenotrophomonas* recommended by the Clinical and Laboratory Standards Institute (14) and the instruction of the CHNENF plate.

### Matrix-assisted laser desorption/ionization time-of-flight mass spectrometry

A small amount of fresh culture of the test strains inoculated on LB agar was spotted onto a target plate. One microliter of formic acid and 1 μL of α-cyano-4-hydroxycinnamic acid matrix solution were overlaid in turn. Detection was performed after drying at room temperature. Two automated microbial mass spectrometry detection systems were used: EXS2000 (Zhongyuan Huiji; Shanghai Jumu Medical Equipment Co., Ltd., China) and Antu Bio (Zhengzhou Antu Bioengineering Co., Ltd., China). Species were judged by the built-in analysis software of the instruments.

### Statistical analysis

Statistical analysis was performed in IBM SPSS Statistics for Windows (v24; IBM Corp., Armonk, NY, USA).

## RESULTS

### Improving the limit of detection of PCR by pre-enrichment

**Fig 1:**
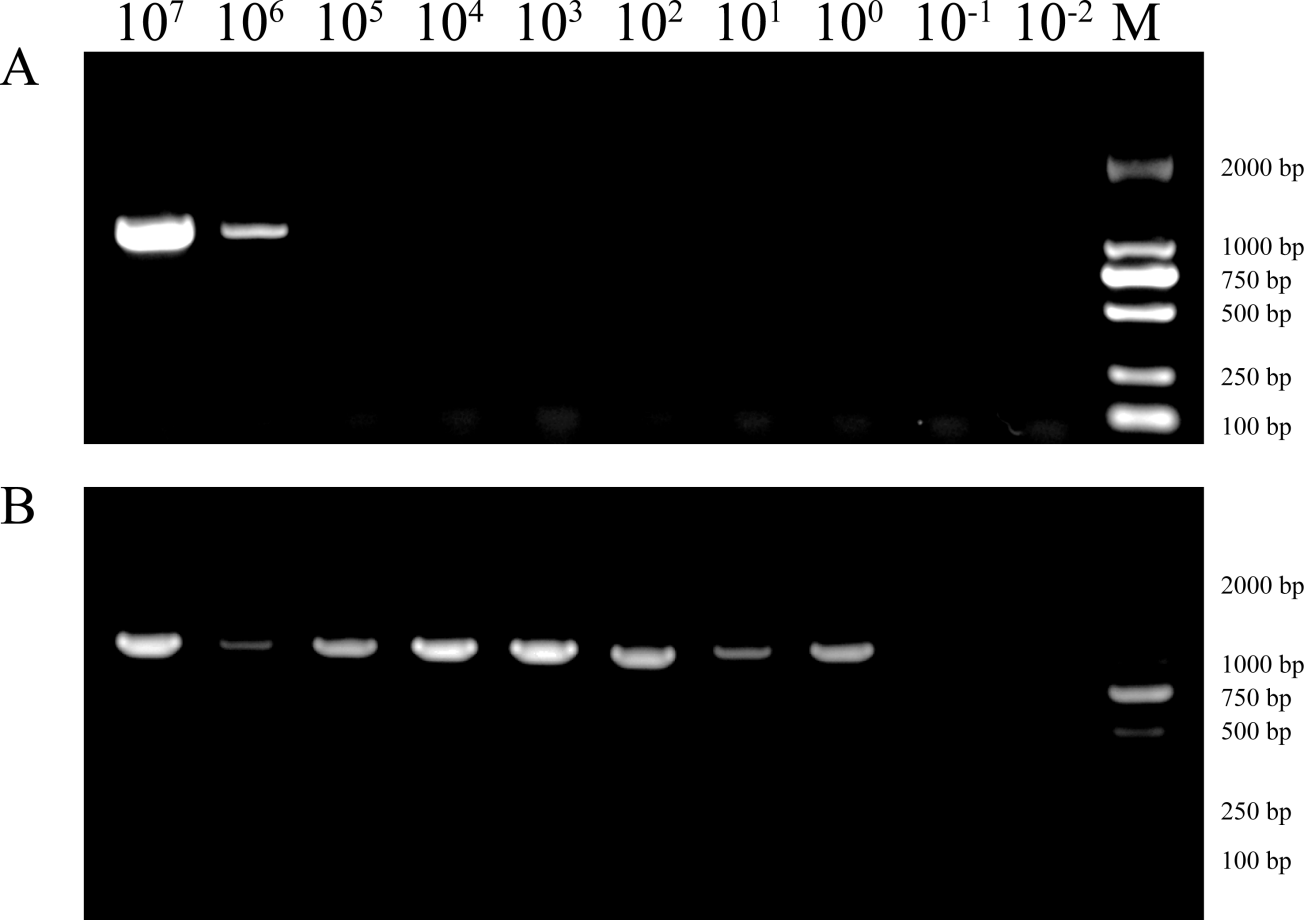
Effect of the pre-enrichment PCR assay. (**A**) Direct PCR amplification of the 10× serially diluted simulated samples. (**B**) PCR amplification of the simulated samples after enrichment culture.

Considering the generally low bacterial load in throat swabs and the limit of detection (LOD) of PCR, we employed an enrichment method in this study to improve the detection rate of *Stenotrophomonas* in throat swab samples. Since throat swab samples contain fewer bacterial species compared to environmental samples such as soil, no antibiotics were added for bacterial inhibition. As shown in Fig. 1 for the simulated samples, pre-enrichment increased the LOD of PCR from 1 × 10^6^ CFU/mL to 1 × 10⁰ CFU/mL, thereby significantly improving sensitivity. The method was thus effective, such that *Stenotrophomonas* could be detected whenever present in the sample.

### *Stenotrophomonas* isolation in the swab samples

During the study period, a total of 143 throat swab samples were collected from 76 male and 67 female patients aged 14–94 years. A *χ*² goodness-of-fit test with 5-year age intervals revealed no statistically significant difference in the distribution of age groups (*χ*² = 14.752, asymptotic significance = 0.323, *P* > 0.05).

Three *Stenotrophomonas* strains were successfully isolated from the PCR-positive samples, yielding an isolation rate of 2.10% (3/143). This rate was statistically significantly lower than the isolation rate of 10.11% observed in TB patients (10) (*χ*² = 7.184, *P* = 0.007).

**Fig 2:**
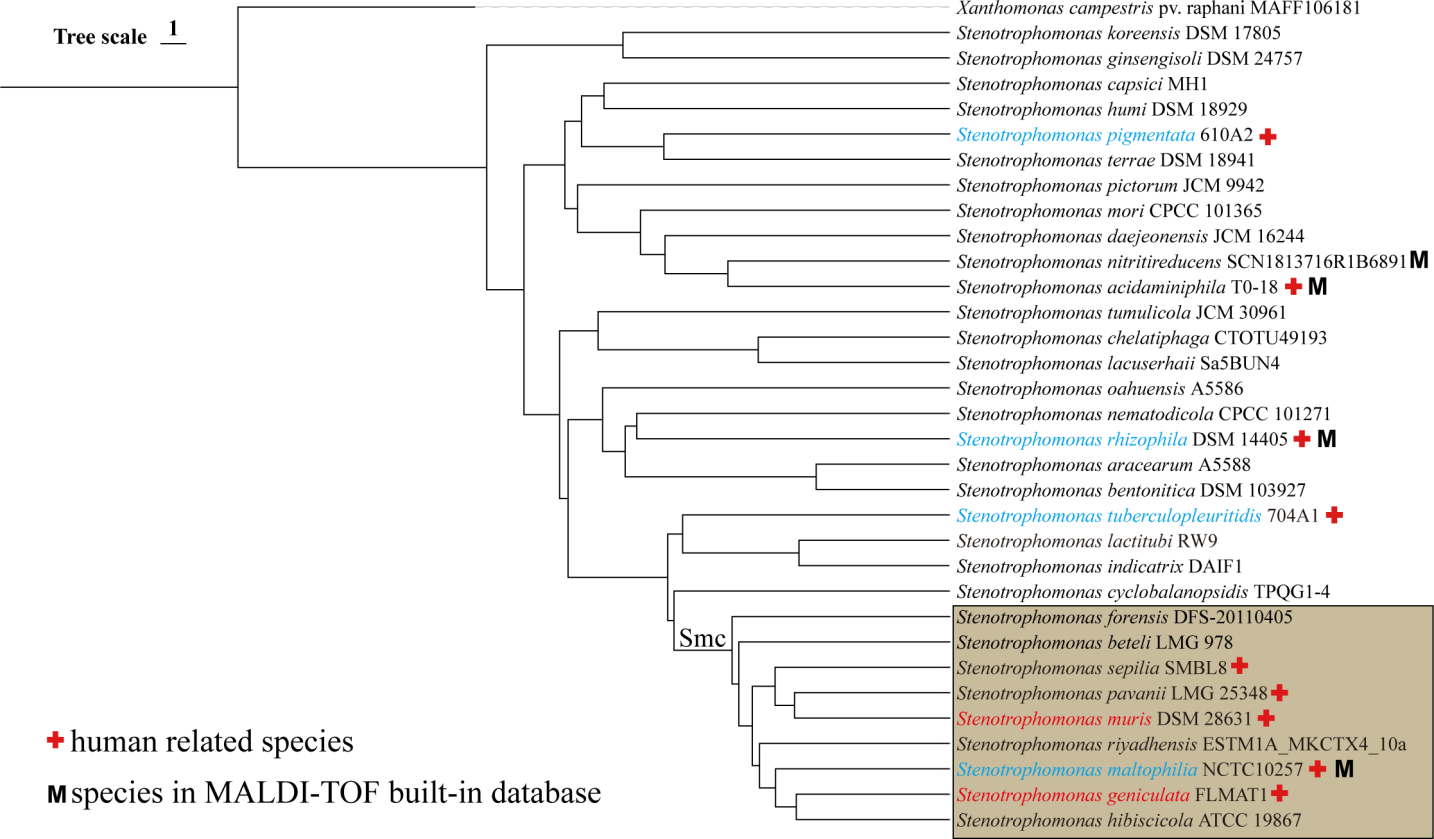
Hierarchical clustering dendrogram based on the skani matrix. Human-related species are marked in red +; species in matrix-assisted laser desorption/ionization time-of-flight (MALDI-TOF) database are marked with M; the names of species identified in this study are written in red, and those identified in TB patients are written in blue.

Species of the three isolate*s* identified based on ANI were *S. geniculate* and *S. muris*, both of which belong to the Smc (Fig. 2). The 16S rRNA sequencing correctly identified the species of C45 (*S. geniculata*) but misidentified *S. muris* as its closest relative, *S. pavanii*. The species identification capabilities of *guaA* and mass spectrometry were even lower, misclassifying all three strains as *S. maltophilia* (Table 1).

**TABLE 1:** Species identification results of the three *Stenotrophomonas* isolates based on *guaA*, 16S rRNA gene sequencing, and ANI

Methods	Strains	Species/genomes with the highest homology	Percent identity (%) value	Coverage (%)
ANI	C45	*S. geniculate* (GCA_034508895.1)	98.13	–^*b*^
	Z98	*S. muris* (GCA_034506535.1)	99.06	–
	Z101	*S. muris* (GCA_034506535.1)	99.06	–
16S rRNA	C45	*S. geniculata* strain BR23 (GCF_031754575.1)	100.00	100
	Z98	*S. pavanii* strain Au-Ste8 (OK178965.1)	99.91	100
	Z101	*S. pavanii* strain Au-Ste8 (OK178965.1)	99.91	100
*guaA*	C45	*S. maltophilia* strain 142 (GCA_023702495.1)	98.18	100
	Z98	*S. maltophilia* strain SVIA2 (GCF_004346945.1)	98.37	100
	Z101	*S. maltophilia* strain SVIA2 (GCF_004346945.1)	98.37	100
MALDI-TOF^*a*^	C45	*S. maltophilia* (ZYAC 06650002A)	2.42	–
	Z98	*S. maltophilia* (FBD 361661)	2.40	–
	Z101	*S. maltophilia* (BJTR 06650001A)	2.33	–

^*a*^ MALDI-TOF, matrix-assisted laser desorption/ionization time-of-flight.

^*b*^ “–” indicates no data.

Whole-genome alignment revealed that strains Z98 and Z101 shared 99.99% aligned bases (4,455,808/4,456,337) with an ANI of 100%. Only seven SNPs and no InDels were detected. This minimal genetic variation indicates that the two strains are clonal and likely originated from nosocomial transmission. This inference is supported by previous studies that have documented a maximum distance of 25 distinct SNPs in grouped patients of presumable patient-to-patient or environment-to-patient transmission (15).

### Case reports of *Stenotrophomonas*-positive patients

Patient C45 was an 84-year-old male, admitted to the hospital due to cough, sputum production, wheezing, and fever lasting for 2 days. Imaging revealed a large patchy consolidation shadow in the upper and middle lobes of the right lung, leading to a suspicion of community-acquired pneumonia. The patient was prescribed intravenous treatment with meropenem combined with linezolid. Laboratory tests showed elevated white blood cell count (16.01 × 10⁹/L), increased absolute neutrophil count (13.91 × 10⁹/L), elevated C-reactive protein (9.64 mg/L), and a positive nucleic acid test for influenza A virus.

Patient Z98 was an 84-year-old female, admitted to the hospital due to abdominal pain lasting for 5 days. The patient had a total bilirubin level of 42.87 μmol/L (↑), with direct bilirubin at 11.85 μmol/L (↑) and indirect bilirubin at 31.02 μmol/L (↑). In conjunction with findings from abdominal and pelvic CT scans, the patient was diagnosed with acute cholecystitis. Markers of infection—including white blood cell count (15.55 × 10⁹/L), absolute neutrophil count (14.91 × 10⁹/L), and C-reactive protein (39.38 mg/L)—were elevated. The patient received percutaneous transhepatic cholangial drainage (PTCD), along with anti-infective therapy involving meropenem and vancomycin.

Patient Z101, a 72-year-old male who had undergone lower extremity arterial stent implantation 8 days prior to admission, was subsequently admitted to the hospital due to fever accompanied by cough and sputum production for 3 days, with dry and moist rales auscultated in both lungs. His white blood cell count and C-reactive protein levels were elevated, and chest CT revealed bilateral pulmonary inflammation. Tests including viral antibodies, a four-item viral panel, fungi, sputum acid-fast staining, and sputum culture with identification all returned negative results. The patient had previously self-administered oral ceftazidime without significant improvement. Following admission, his body temperature normalized after receiving anti-infective therapy with moxifloxacin.

### Antibiotic resistance profiles of the isolates

The AST results of 17 drugs showed that all tested strains were highly resistant to penicillins, carbapenems, and polymyxins antibiotics but sensitive to quinolones, tigecycline, chloramphenicol, and trimethoprim-sulfamethoxazole (TMP-SMX) (Table 2). The AST profiles of *S. muris* Z98 and Z101 were largely consistent with that of *S. maltophilia* 11066. Notably, however, they demonstrated susceptibility to ceftazidime and ceftazidime/avibactam. The AST profile of *S. geniculate* strain C45 is highly unique, exhibiting resistance only to β-lactam antibiotics and colistin.

**TABLE 2:** The AST results of the three *Stenotrophomonas* isolates and reference strain 11066

Class	Drug	Concentration tested (μg/mL)	MIC (μg/mL)				Breakpoints (μg/mL)
			C45	Z98	Z101	11,066	
Penicillins	Ampicillin	2–32	>32	>32	>32	>32	≥32
	Ampicillin/sulbactam	2/1–32/16	>32/16	>32/16	>32/16	>32/16	≥32/16
Carbapenems	Meropenem	0.12–2.0	>2	>2	>2	>2	≥4
	Ertapenem	0.25–8.0	>8	>8	>8	>8	≥2
Cephalosporins	Ceftazidime	0.25–16.0	>16	4	8	>16	≥32^*a*^
	Ceftazidime/avibactam	0.25/4.0–8/4	>8/4	2/4	2/4	>8/4	≥16/4
	Cefotaxime	0.25–16.0	>16	>16	>16	>16	≥4
Macrolides	Azithromycin	2–64	<2	64	32	64	≥32
Quinolones	Ciprofloxacin	0.015–2.0	0.12	2	2	2	≥8^*a*^
	Nalidixic acid	4–32	<4	8	8	8	≥32
Aminoglycosides	Amikacin	4–64	<4	64	>64	>64	≥64
	Streptomycin	8–32	16	>32	>32	>32	≥32
Tetracyclines	Tetracycline	1–16	<1	16	16	16	≥16
	Tigecycline	0.25–8.0	<0.25	0.5	0.5	0.5	≥8
Chloramphenicols	Chloramphenicol	4–32	<4	16	16	16	≥32^*a*^
Polymyxins	Colistin	0.25–8.0	>8	>8	>8	>8	≥4
Sulfonamides	TMP-SMX	0.5/9.5–8/152	<0.5/9.5	<0.5/9.5	<0.5/9.5	<0.5/9.5	≥4/76^*a*^

^*a*^ Clinical and Laboratory Standards Institute-recommended breakpoints; drugs determined to be resistant according to antimicrobial breakpoints are in gray boxes.

## DISCUSSION

All of the routine clinical tests of these three patients failed to detect *Stenotrophomonas*, suggesting it is a highly covert pathogenic factor. In this study, in addition to two patients with pulmonary infections, *Stenotrophomonas* strain Z98 was isolated from a patient with acute cholecystitis. Animal infection models have shown that *Stenotrophomonas* can be isolated from the spleen as early as 4 h after intranasal inoculation, underscoring its strong tissue invasiveness and hematogenous dissemination capacity (16, 17). Therefore, Z98 may have either colonized the pharynx directly via airborne transmission or reached the pharynx through hematogenous spread from the gallbladder following PTCD. Regardless of the route, these results suggest that pharyngeal swabs could serve as a valuable diagnostic indicator for *Stenotrophomonas*, even when the primary site of infection or colonization is in non-respiratory tissues. In addition to its own pathogenicity, *Stenotrophomonas* can impact the disease progression of other co-infecting pathogens. For example, apart from its well-known role in cystic fibrosis, the bacterium can, during co-infections, exacerbate influenza virus infection through the action of the bacterial protease elastase, which cleaves and activates the hemagglutinin of these viruses (18). Moreover, whether during colonization or infection, *Stenotrophomonas* can produce virulence factors, such as hyaluronidase, hemolysin, and keratinase, within the host, which may cause host damage. Therefore, improving the detection rate of this pathogen and providing clinicians with indications of its presence are highly necessary, especially in critically ill patients and those with unexplained etiologies.

A summary of three *Stenotrophomonas*-positive cases revealed that advanced age (>70 years, mean age 80 years) and iatrogenic factors were their common characteristics. Iatrogenic factors included invasive medical procedures (e.g., vascular surgery and PTCD), repeated hospitalizations, and exposure to broad-spectrum antimicrobials. All patients had received combination antibiotic therapy, including regimens such as meropenem plus linezolid or vancomycin, and ceftazidime plus moxifloxacin. These are essentially the same high-risk factors associated with the developing *S. maltophilia* infection but differ from the characteristics of TB patients positive for *Stenotrophomonas*. The latter are primarily in the 24–38 age group; none have a history of interventional therapy; and although they have received multidrug anti-TB treatment, their regimens did not include β-lactams (such as cephalosporins) or TMP-SMX, which are high risk factors for *S. maltophilia* (8, 10). Furthermore, the strains isolated from inpatients in this study identified as *S. muris* and *S. geniculate* both belong to the Smc despite not being *S. maltophilia* itself. However, some strains isolated from TB patients belonged to species outside the Smc and demonstrated greater genetic diversity (10). These differential characteristics between *Stenotrophomonas*-positive TB and no-TB patients will be validated in subsequent, larger-scale surveillance studies.

*S. muris* is a novel species within the *Stenotrophomonas* genus, first identified in 2022 (19) and reported as a potential human pathogen with strong virulence and antibiotic resistance, associated with bloodstream infection (20). Our findings further suggest that it may play a significant role in hospital-acquired infections. The results also revealed that in routine clinical microbiology identification, both *S. muris* and *S. geniculate* were misidentified by matrix-assisted laser desorption/ionization time-of-flight (MALDI-TOF) as *S. maltophilia*, despite all producing identification scores of ≥2.0, which typically guarantees reliable species-level identification. A similar issue was documented in Liu et al.’s report (20). Currently, all available integrated MALDI-TOF system databases such as Bruker_MSP library contain at most five species of the genus *Stenotrophomonas*, namely, *S. acidaminiphila*, *S. maltophilia*, *S. rhizophila*, *S. nitritireducens*, and *S. pictorum* (the latter is absent from certain databases) (Fig. 2). The species coverage is severely insufficient compared to the 32 named and 116 ANI *Stenotrophomonas* species. What’s worse, the commonly used rapid identification methods based on conservative single-gene sequencing, such as 16S rRNA and *guaA*, also exhibit a very high error rate in identifying *Stenotrophomonas* species. Species misidentification of the isolates has several negative impacts: it hinders the discovery of emerging pathogens, such as *S. muris* and *S. tuberculopleuritidis*, and leads to an exclusive focus on *S. maltophilia* and underestimation of the clinical importance of species missing from the MALDI-TOF database and misinterpretations of their characteristics. Thus, alongside the mass spectral database expansion, establishing alternative rapid molecular methods for *Stenotrophomonas* species with ANI-equivalent resolution is essential.

Currently, susceptibility data for *S. muris* and *S. geniculate* remain very limited. This study demonstrated that the tested strains, including the control strain *S. maltophilia* 11066, were generally susceptible to quinolones, tigecycline, and chloramphenicol, with particularly high susceptibility to TMP-SMX. The two *S*. *muris* strains, Z98 and Z101, were susceptible to both ceftazidime and ceftazidime/avibactam, a finding consistent with Liu’s report. However, their susceptibility to TMP-SMX differed from that of the *S. muris* strains described by Liu et al. (20). The *S. geniculate* strain C45 showed higher susceptibility to clinical antimicrobials compared to the Japanese isolates in Hase et al.’s study (21) and could not be classified as an MDR strain. Therefore, the prevailing notion that *Stenotrophomonas* or *S. maltophilia* is intrinsically resistant to β-lactams, carbapenems, fluoroquinolones, tetracyclines, chloramphenicol, macrolides, and TMP-SMX warrants reevaluation. Nevertheless, the resistance profile of *S. geniculate* strain C45 to β-lactams and colistin is as concerning as that of some MDR bacteria, as it effectively negates several key classes of first-line drugs, including a last-resort option, thereby complicating the management of polymicrobial infections. In addition, while the post-treatment detection of meropenem-resistant strains C45 and Z98 in the throat is expected, it is notable that Z101—despite being susceptible to both ceftazidime and moxifloxacin—was still able to colonize the throat following therapy. This is presumably due to insufficient local drug concentrations in the throat.

### Conclusions

This study employed a sensitive pre-enrichment PCR assay to detect *Stenotrophomonas* in throat swabs, demonstrating its value in alerting clinicians to this covert pathogen, even in patients hospitalized for non-respiratory conditions. The risk factors identified were distinct from those in *Stenotrophomonas*-positive TB patients. Furthermore, our findings call into a critical issue of the misidentification of species by routine clinic microbiological methods, which has perpetuated the misconception and underestimation about the genus *Stenotrophomonas* and its species.
